# Supplementary material for: Preanalytical considerations for clinical assays of circulating human miRNA-451a, miRNA-423-5p and miRNA-199a-3p for diagnostic purposes
Source: PLoS One. 2024 May 20;19(5):e0303598. doi: 10.1371/journal.pone.0303598 (PMC11104614; doi:10.1371/journal.pone.0303598)
Supplement: Attachment — Submitted filename: Response to Reviewers.docx [file pone.0303598.s001.docx]

**Response to Reviewers**

RE: Manuscript # PONE-D-24-05265, titled “Preanalytical considerations for clinical assays of circulating human miRNA-451a for diagnostic purposes”.

April 10, 2024.

Professor Himanshu Gupta

Academic Editor

PLOS ONE

Dear Professor Gupta,

We would like to take this opportunity to thank you for considering our manuscript for peer review, and the opportunity for a revised submission. Your valuable editorial comments have helped us improve our article as per PLOS ONE format. We welcome all suggestions and comments made by both the reviewers as equally important and have tried to address them all with incorporated necessary changes in our revised submission. Below is the pointwise response in brief, to all queries/comments received in your review report.

Editors’ comments and reply to editors’ comments.

Please include the following items when submitting your revised manuscript:

- A rebuttal letter that responds to each point raised by the academic editor and reviewer(s). You should upload this letter as a separate file labeled 'Response to Reviewers'.

Rebuttal letter enclosed.

- A marked-up copy of your manuscript that highlights changes made to the original version. You should upload this as a separate file labeled 'Revised Manuscript with Track Changes'.

Uploaded the track-change version of the revised manuscript.

- An unmarked version of your revised paper without tracked changes. You should upload this as a separate file labeled 'Manuscript'.

Final revised version without tracked changes, uploaded.

If you would like to make changes to your financial disclosure, please include your updated statement in your cover letter. Guidelines for resubmitting your figure files are available below the reviewer comments at the end of this letter.

Journal Requirements: When submitting your revision, we need you to address these additional requirements.

1. Please ensure that your manuscript meets PLOS ONE's style requirements, including those for file naming. The PLOS ONE style templates can be found at

[https://journals.plos.org/plosone/s/file?id=wjVg/PLOSOne_formatting_sample_main_body.pdf](https://urldefense.com/v3/__https:/journals.plos.org/plosone/s/file?id=wjVg*PLOSOne_formatting_sample_main_body.pdf__;Lw!!CEjAKCqGPSl6!oqCzz8RBITQu4PsGCZCijm2jjfD9Iwv1Vkeiz6e0xzqEUTfYRwXjoRwsDWLehraBwieAivj_uUWqDOG0wGyjAy63$) and 
[https://journals.plos.org/plosone/s/file?id=ba62/PLOSOne_formatting_sample_title_authors_affiliations.pdf](https://urldefense.com/v3/__https:/journals.plos.org/plosone/s/file?id=ba62*PLOSOne_formatting_sample_title_authors_affiliations.pdf__;Lw!!CEjAKCqGPSl6!oqCzz8RBITQu4PsGCZCijm2jjfD9Iwv1Vkeiz6e0xzqEUTfYRwXjoRwsDWLehraBwieAivj_uUWqDOG0wI7hgwyw$)

- The manuscript has been formatted following PLOS One requirements.

2. We noticed you have some minor occurrence of overlapping text with the following previous publication(s), which needs to be addressed:

[https://www.preprints.org/subject/browse/medicine_and_pharmacology/clinical_medicine_130?](https://urldefense.com/v3/__https:/www.preprints.org/subject/browse/medicine_and_pharmacology/clinical_medicine_130?__;!!CEjAKCqGPSl6!oqCzz8RBITQu4PsGCZCijm2jjfD9Iwv1Vkeiz6e0xzqEUTfYRwXjoRwsDWLehraBwieAivj_uUWqDOG0wKX_V1jJ$)

In your revision ensure you cite all your sources (including your own works), and quote or rephrase any duplicated text outside the methods section. Further consideration is dependent on these concerns being addressed.

- The overlapping text is a preprint (unpublished) generated from a past submission to a different journal, as some journals encourage authors to upload a preprint during submissions. However, our manuscript was rejected by that journal and for this current PLOS ONE submission, we conducted additional experiments and updated extensively, comparing all three miRNAs for this study. This is also reflected within the revised title. As suggested, our current revised submission to PLOS ONE has citations updated and duplicated texts have been either omitted or rephrased.

3. We note that the grant information you provided in the ‘Funding Information’ and ‘Financial Disclosure’ sections do not match.

When you resubmit, please ensure that you provide the correct grant numbers for the awards you received for your study in the ‘Funding Information’ section.

- The funding information has been checked and grant number updated.

4. Thank you for stating the following financial disclosure:

"This research was funded by a research grant from Ryan Foundation given to MRF."

Please state what role the funders took in the study.  If the funders had no role, please state: "The funders had no role in study design, data collection and analysis, decision to publish, or preparation of the manuscript." If this statement is not correct you must amend it as needed.

Please include this amended Role of Funder statement in your cover letter; we will change the online submission form on your behalf.

- The revised Financial Disclosures statement has been added separately. “This research was funded by a research grant (RFLG-2019-2023-0003) from the Ryan Foundation to MRF. The funders had no role in study design, data collection and analysis, decision to publish, or preparation of the manuscript.”

5. Thank you for stating the following in the Acknowledgments Section of your manuscript:

"We wish to thank Dr. Dominic Cosgrove, Center for Sensory Neuroscience, Boys Town National
Research Hospital, Omaha NE USA for his advice and help during this project. We gratefully
acknowledge the assistance from Rebecca Cash for IRB application. We want to thank Dan
Meehan, Center for Sensory Neuroscience for his help. This research was funded by a research
grant from the Ryan Foundation to MRF."

Please note that funding information should not appear in the Acknowledgments section or other areas of your manuscript. We will only publish funding information present in the Funding Statement section of the online submission form.

Please remove any funding-related text from the manuscript and let us know how you would like to update your Funding Statement. Currently, your Funding Statement reads as follows:

"This research was funded by a research grant from Ryan Foundation given to MRF."

Please include your amended statements within your cover letter; we will change the online submission form on your behalf.

- The funding information has now been sated under the assigned section separate from acknowledgements.

6. PLOS requires an ORCID iD for the corresponding author in Editorial Manager on papers submitted after December 6th, 2016. Please ensure that you have an ORCID iD and that it is validated in Editorial Manager. To do this, go to ‘Update my Information’ (in the upper left-hand corner of the main menu), and click on the Fetch/Validate link next to the ORCID field. This will take you to the ORCID site and allow you to create a new iD or authenticate a pre-existing iD in Editorial Manager. Please see the following video for instructions on linking an ORCID iD to your Editorial Manager account: [https://www.youtube.com/watch?v=_xcclfuvtxQ](https://urldefense.com/v3/__https:/www.youtube.com/watch?v=_xcclfuvtxQ__;!!CEjAKCqGPSl6!oqCzz8RBITQu4PsGCZCijm2jjfD9Iwv1Vkeiz6e0xzqEUTfYRwXjoRwsDWLehraBwieAivj_uUWqDOG0wICBoW8o$)

- The ORCID iD has been linked and validated in Editorial manager.

7. Please include captions for your Supporting Information files at the end of your manuscript, and update any in-text citations to match accordingly. Please see our Supporting Information guidelines for more information: [http://journals.plos.org/plosone/s/supporting-information.](https://urldefense.com/v3/__http:/journals.plos.org/plosone/s/supporting-information.__;!!CEjAKCqGPSl6!oqCzz8RBITQu4PsGCZCijm2jjfD9Iwv1Vkeiz6e0xzqEUTfYRwXjoRwsDWLehraBwieAivj_uUWqDOG0wH7sOsSf$)

- All captions/supporting figures (now included in results) have been shifted to the main text.

1. 8. We notice that your supplementary figures are uploaded with the file type 'Figure'. Please amend the file type to 'Supporting Information'. Please ensure that each Supporting Information file has a legend listed in the manuscript after the references list.
   [Note: HTML markup is below. Please do not edit.]

- Considering valuable suggestions received from both reviewers, we now have shifted all of previous supplement results/figures describing the other two miRNAs to the main manuscript-text, and relabeled them in the order cited.

Response to reviewer 1

**Reviewer #1:** The manuscript is related to preanalytical factors that can affect circulating miRNAs, an important issue to be considered, especially due to higher variability in the assays.

- Thank you for an extensive review of our submission to PLOS ONE, recognizing the importance of this study in context of miRNA stability during preanalytical phase, pertaining to high variability observed in clinical assays.

In order to publish this article, some corrections must be made:
Objectives
1. The authors should clarify the objectives, as the miRNA-451a was considered the main miRNA of this work (considering the title), but all over the text and even in the last phrase of introduction other miRNAs are mentioned. Are the results of other miRNAs (miRNA-423-5p and miRNA-199a-3p) relevant to the manuscript? If so they should be addressed in the objectives and introduction.

- Thank you for pointing out this weakness in our manuscript. By changing the title, objectives in the abstract and the introduction we tried to address this issue. The title, objectives in the abstract and the introduction was changed to include all three miRNAs. Considering the other two miRNAs equally important, we have updated the manuscript placing these miRNAs along with the miRNA-451a in all sections.

2. Introduction
   Information related to miRNA-423-5p and miRNA-199a-3p should be included in the introduction instead of results.

- As per reviewers’ suggestion, the other two miRNAs (miR-423/199a) now are also highlighted in the Intro/Discussion sections, with a revised results section.

3. Methods and Materials
   The number (n) of blood samples used to extract miRNA from whole blood, PRP, PFP should be listed at materials and methods. It is not clear all over the text. Was this “n” enough to support the results?

- As per your suggestion Methods and materials section (Blood samples section) was modified to include number of blood donors used for each experiment.

4. Results
   The information from “MiRNA-423-5p is known for its high abundance in plasma and has been reported as an endogenous control for the quantification of circulating miRNAs in certain cancers” and “MiRNA-199a-3p is another important miRNA that is highly abundant in blood, shown as a promising diagnostic and prognostic marker in glioma” should be moved to introduction.

- As per your suggestion we moved that information from results section to introduction.

Are there any result related to effect of hemolysis on miRNA-423-5p and miRNA-199a-3p?

- Yes, we have modified Fig 1 to include the results for miRNA-423-5p and miRNA-199a-3p.

In case the miRNA-423-5p and miRNA-199a-3p will be included in the objectives, the supplemental figures should be moved to results, maybe including different bar colors to the previous ones.

- As per your suggestion we moved all supplemental Figures to main text.

Conclusion
It is important to include a conclusion related to miRNA-423-5p and miRNA-199a-3p results or that the preanalytical interferences may vary according to miRNA used.

- This section is now updated, with all three miRNAs compared, as preanalytical factors may vary across miRNA families. Since miRNAs stability can be inherently different due to their specific origin or preprocessing events during maturation and function, we emphasize that levels of disease specific/individual miRNAs should therefore be interpreted with caution.

**Reviewer #2:**

Report on "Preanalytical Considerations for Clinical Assays of Circulating Human miRNA-451a for Diagnostic Purposes"
The manuscript investigates preanalytical factors affecting the accurate quantification of miRNA-451a in human blood samples, crucial for its diagnostic potential. Key findings include the predominant localization of miRNA-451a in red blood cells (RBCs) and significant hemolysis impact on plasma concentration. Storage conditions also play a role, with RT and 4ºC affecting platelet-rich plasma (PRP) and platelet-free plasma (PFP) stability, while -80ºC maintains stability up to 7 days.

- Thank you for an extensive review of our submission to PLOS ONE, recognizing the importance of this study in context of miRNA stability during preanalytical phase, pertaining to high variability observed in clinical assays.

Review:
Sample Size Clarity: The manuscript utilizes normal healthy samples; however, the number of samples is not specified. Clear reporting of the sample size is essential for result interpretation and study validity.

As per your suggestion Methods and materials section (Blood samples section) was modified to include number of blood donors used for each experiment.

Enhanced Study Design: To strengthen the study's impact, it is suggested to consider incorporating different patient or disease samples for comparison with controls. This addition would provide valuable insights into the specificity and diagnostic potential of miRNA-451a across various health conditions.

- We understand extending our analysis on patient samples may enhance study’s impact. This remains our future goal with ongoing biomarker research. Our current IRB approval is only for normal blood donors and not for patient samples. We hope to continue this project with patient samples in near future with a new IRB protocol.

Conclusion: The study offers valuable insights into preanalytical considerations for miRNA-451a quantification, emphasizing the need for careful handling to ensure accurate results. Addressing the highlighted points could further enhance the robustness and applicability of the findings.

- Thank you for your valuable comments. We hope that the revisions we made to this version of our manuscript enhanced the applicability of our findings in laboratory medicine.
