# Supplementary material for: Preanalytical considerations for clinical assays of circulating human miRNA-451a, miRNA-423-5p and miRNA-199a-3p for diagnostic purposes
Source: PLoS One. 2024 May 20;19(5):e0303598. doi: 10.1371/journal.pone.0303598 (PMC11104614; doi:10.1371/journal.pone.0303598)
Supplement: Attachment — Submitted filename: Recomendation letter.docx [file pone.0303598.s002.docx]

Thank you for informing me. The modifications made to the paper will undoubtedly enhance its quality and relevance. Looking forward to its publication and contributing to the field of preanalytical considerations for clinical assays of circulating human miRNA.
